# Changing Levels of Social Engagement with Gay Men Is Associated with HIV Related Outcomes and Behaviors: Trends in Australian Behavioral Surveillance 1998–2020

**DOI:** 10.1007/s10508-022-02310-x

**Published:** 2022-06-07

**Authors:** Curtis Chan, Benjamin R. Bavinton, Garrett E. Prestage, Timothy R. Broady, Limin Mao, John Rule, Ben Wilcock, Martin Holt

**Affiliations:** 1 Kirby Institute, UNSW Sydney, Level 6 Wallace Wurth Building High Street, Kensington, Sydney, NSW 2052 Australia; 2 Centre for Social Research in Health, UNSW Sydney, Sydney, Australia; 3 National Association of People with HIV Australia, Sydney, Australia; 4 Australia Federation of AIDS Organisations, Sydney, Australia

**Keywords:** Men who have sex with men, HIV, Gay community, Sexual health, Social networks, Sexual orientation

## Abstract

Changes to how gay, bisexual, and other men who have sex with men (GBM) connect with each other and with their communities have implications for HIV prevention. Social engagement with gay men (defined as having friends who are gay men and spending time with them) has been associated with HIV related outcomes over time among Australian GBM. Using data collected in national, repeated, cross-sectional surveys of GBM between 1998 and 2020 (*N* = 161,117), analyses of trends in the prevalence of gay social engagement (GSE) in Australia were conducted using linear regression. To assess changing associations with GSE at different time points in the HIV epidemic, three cross-sectional analyses were conducted on factors associated with high and low GSE in 1999/2000, 2009/2010, and 2019/2020 using bivariate and multivariable logistic regression. GSE (scored from 0 to 7) declined among all participants from 4.76 in 1998 to 4.04 in 2020 (*p* < 0.001) with a steeper decline among GBM aged under 25 years from 4.63 in 1998 to 3.40 in 2020 (*p* < 0.001). In all timepoints, high GSE was associated with older age, being university educated, full time employment, identifying as gay, recent HIV testing, and PrEP uptake. While mostly associated with protective behaviors, high GSE was also associated with some practices that may put GBM at risk of HIV infection such as drug-enhanced sex and group sex in the most recent timepoint. Changing levels of GSE have implications for health promotion among GBM, particularly how to engage GBM less connected to gay social networks.

## Introduction

HIV continues to disproportionately affect gay and bisexual men, and other men who have sex with men (GBM), both in Australia and internationally (Kirby Institute, 2020; World Health Organization, 2020). Community mobilization of and engagement with GBM has been central to HIV prevention efforts in Australia for decades (Power, 2011; Sendziuk, 2003) and gay community engagement is recognized as important in national and state HIV strategies (Department of Health, 2018; Department of Health & Human Services, 2017; NSW Health, 2019). Due to societal shifts in attitudes towards homosexuality and greater social acceptance, GBM’s social networks have diversified with some evidence suggesting that younger GBM are becoming less reliant on gay community for social support (Lelutiu-Weinberger et al., 2013; Lewis et al., 2015). Socializing has also been affected by changes in the way that GBM meet each other, with a greater reliance on dating and hook-up apps and reduced attendance at physical spaces like gay bars (Buzi et al., 2021; Hubbard et al., 2016).

Previous work has found an association between an individual’s social network and HIV prevention outcomes. Individuals are more likely to believe they are at a high risk of HIV acquisition if they also believe members of their social network are at high risk (Koku & Felsher, 2020). Among young black GBM, social support from other young black GBM has been found to be associated with higher levels of HIV testing (Lauby et al., 2012; Scott et al., 2014). Among young GBM, involvement in gay community networks and network expertise (i.e., knowing someone who is knowledgeable about HIV) has been associated with seeking out information about HIV (Veinot et al., 2013). Recently, HIV pre-exposure prophylaxis (PrEP) uptake has been associated with GBM and women having larger peer networks (Johnson et al., 2021; Kuhns et al., 2017), GBM knowing more PrEP users (Holt et al., 2019), and greater social support among young GBM and transgender women (Chen et al., 2020). This evidence is consistent with various models of health behavior that suggest that social norms and observing or modelling behavior in one’s social network influence HIV-related health behaviors (Kaufman et al., 2014).

There is longstanding evidence that social engagement between gay men is associated with HIV prevention outcomes and sexual behavior, but these associations have changed over time. The Gay Social Engagement (GSE) scale developed by Kippax et al. (1992) measured the social involvement of Australian gay men with other gay men and was part of a broader set of scales measuring attachment to and involvement in gay community activities in the first decade of the HIV epidemic. It was based on an assumption that social connectedness and social contact with other gay men would enable the exchange of information about HIV, learning about safe sex, and modeling of preventive behavior. It focused on behavior (what people did and where they spent time) rather than an affective or cognitive feeling of attachment to gay community. A truncated version of the scale containing two items with the highest item-total correlation has been used frequently in research and is used as a proxy for the longer measure (Hammoud et al., 2017b). While the two-item measure does not comprehensively capture the elements of gay men’s social networks, it has been shown to be consistently associated with HIV-related outcomes.

In the early 1990s, higher levels of social engagement between gay men were associated with the adoption of condom use in response to HIV (Kippax et al., 1992, 1995). Later in the epidemic, higher GSE became associated with the likelihood of reporting condomless anal sex with casual partners (Fergus et al., 2009; Foster-Gimbel et al., 2020; Van De Ven et al., 2002), a higher number of sexual partners (Zablotska et al., 2012), binge-drinking (Fisher et al., 2014), illicit drug use (Bui et al., 2018; Hammoud et al., 2017a, 2018), and not consistently using HIV risk reduction strategies, such as strategic positioning or withdrawal, during condomless sex (Kolstee et al., 2020). While GSE appears to have become associated with a number of risk factors for HIV in the second and third decades of the HIV epidemic, GSE also continues to be associated with protective factors including HIV testing (Bavinton et al., 2020; Keen et al., 2020; Lee et al., 2020; Van de Ven et al., 2000; Zablotska et al., 2012) and PrEP uptake (Keen et al., 2020). This suggests that the association between GSE and various HIV prevention behaviors has changed over time, and that GSE has been associated both with HIV risk and protective factors.

Behavioral surveillance data in Australia indicates that even in samples of GBM, who were largely recruited from gay venues and events and appeared to be highly community engaged, GSE declined between 1998 and 2009 (Zablotska et al., 2012). It was suggested that GSE may have declined because GBM had become less reliant on each other for social support, and more integrated into heterosexual networks, in the context of more supportive social attitudes towards minority sexualities. There had also been a shift from meeting and socializing exclusively in physical locations to a mixture of online and offline locations (Card et al., 2018; Hull et al., 2016). Since this earlier research, there have been significant public debates about and ongoing shifts in attitudes towards minority sexualities in Australia, evident in the public debate about and passing of marriage equality legislation in 2017, and current debates about anti-discrimination laws. This suggests that an updated analysis of GSE is warranted, as GBM’s social networks and patterns of socializing may have continued to change.

Understanding long-term trends in GSE among GBM and its relationship to HIV-related behaviors is important for HIV prevention because community mobilization and engagement have remained central components of the HIV response in Australia and similar countries (Lewis et al., 2015; Power, 2011; Sendziuk, 2003; Trapence et al., 2012). The early rollout of PrEP in Australia relied on gay community mobilization, and there have been lower levels of PrEP uptake by GBM less connected to gay networks (Grulich et al., 2018; Holt et al., 2019). If GSE is declining but implementation remains focused on GBM’s social networks, this suggests that PrEP rollout may become more difficult. More broadly, understanding long-term changes in GBM’s social networks and the relationship of GSE to HIV-related behaviors is useful to consider how mobilization and engagement strategies need to evolve and change, as they have done throughout the epidemic. The aims of this analysis were to assess the changes in GSE over time, and determine factors associated with high and low GSE over two decades, including in the most recent period. Based on previous research (Zablotska et al., 2012), we expected GSE to have further declined between 1998 and 2020, that high GSE would continue be associated with protective behaviors like HIV testing and PrEP, but that it may also be associated with risk-taking behaviors like condomless sex, a higher number of sexual partners, and drug use.

## Method

### Participants and Procedure

The methods of the Gay Community Periodic Surveys (GCPS) have been previously described (Holt et al., 2017; Zablotska et al., 2011). Briefly, the GCPS are repeated cross-sectional surveys which recruit GBM at gay events, bars, clubs, sex-on-premises venues, clinics and online. Online recruitment has been included since 2014. Participants were eligible if they are aged 18 years or over in face-to-face recruitment or 16 years or over in online recruitment, identify as men (inclusive of cisgender and transgender men), have had sex with a man in the past five years or identify as gay or bisexual, and live in Australia. This analysis includes data from 1998 to 2020.

### Measures

The core questionnaire measures have been previously described (Holt et al., 2017; Zablotska et al., 2011). Two questions measured social engagement with gay men: “How many of your friends are gay or homosexual men?” (None, A few, Some, Most, All; scored 0–4) and “How much of your free time is spent with gay or homosexual men?” (None, A little, Some, A lot; scored 0–3). The responses to these questions were summed to form an 8-point scale from 0 to 7. Scores from 0 to 3 were categorized as low GSE (having few gay friends and spending little time with gay men) and 4–7 as high GSE (having at least some gay friends and spending some or a lot of your time with gay men; Zablotska et al., 2012). The GSE scale’s internal consistency (reliability) during the period of analysis was *α* = 0.67–0.77. Demographic characteristics such as age, country of birth sexual identity, the highest level of education attained, employment, and relationship status were included in analyses. Age was included as a continuous variable. Participants reported their sexual identity, education, and employment by selecting from a list of options, or were able to write in their own answer for country of birth, ethnicity, and sexual identity, as described in Zablotska et al. (2011) and Broady et al. (2021). Participants’ sexual identities were categorized as “gay,” “bisexual” or another sexual identity. As a range of independent variables were included in these analyses, variables with a large number of response categories were generally dichotomized to simplify analyses and reporting. Participants were dichotomized into groups based on whether they were born in Australia or not, whether they identified as Anglo-Australian or not, whether they identified as Aboriginal or Torres Strait Islander or not, had a university degree or not, or were in full time employment or not. HIV status (and PrEP use) was accounted for in the analyses by categorizing participants as HIV-negative not on PrEP, HIV-negative on PrEP, HIV-positive, and participants who did not know their HIV-status or were untested. Recent HIV testing was defined as participants receiving an HIV test in the previous twelve months or not. Sexual behavior and drug use in the previous six months were also included in the analyses. Participants were dichotomized into groups based on whether they had 11 or more recent male sexual partners, had engaged in condomless anal intercourse with casual partners (CLAIC) or not, condomless anal intercourse with regular partners (CLAIR) or not, engaged in group sex, engaged in recent injecting drug use, or recently used drugs for sex. In Australia, sex with casual male partners has been identified as a greater source of HIV risk than sex with regular male partners, and the use of drugs for sex has previously been found to be an indicator of increased risk (Down et al., 2017; Holt et al., 2015). Participants were also asked where they had met their male sexual partners in the previous six months at gay bars, through mobile applications (e.g., Grindr, Scruff, etc.) or through the internet. Participants were also dichotomized into those who had received any sexual transmitted infection (STI) diagnosis in the previous twelve months or not.

### Analyses

Linear regression analyses were performed to assess trends over time with GSE as the dependent measure and year as the independent variable. From our previous work, younger GBM are known to have lower levels of GSE (Chan et al., 2020). The trend analyses were repeated, stratifying by age (under 25 years vs. 25 years and over). Cross-sectional analyses were also performed at three timepoints (1999/2000, 2009/2010 and 2019/2020). As the survey is conducted biennially in some states, each timepoint represents a national sample in that the most recent round in each state was included in the cross section. Bivariate logistic regression was conducted at each timepoint comparing the characteristics of those with low and high GSE on variables that have been consistently collected during 1999–2020. For the 2019/2020 timepoint, additional bivariate and multivariable logistic regression models were performed to compare participants with low GSE and high GSE using the wider range of variables available in this period e.g., including PrEP use. Participants with missing data that could not be recorded in the multivariable analysis were excluded. Variables with a significant association at the bivariate level (*p* < 0.05) were block entered in the multivariable model. Multicollinearity was assessed using the variance inflation factor (VIF) where a VIF of over 10 indicates variables are collinear (Chatterjee, 1991). Unadjusted and adjusted odds ratios, 95% confidence intervals and *p* values are reported. Analyses were conducted in Stata 14.2 (StataCorp, College Station, Texas, USA).

## Results

### Trends in Gay Social Engagement

**Fig. 1:**
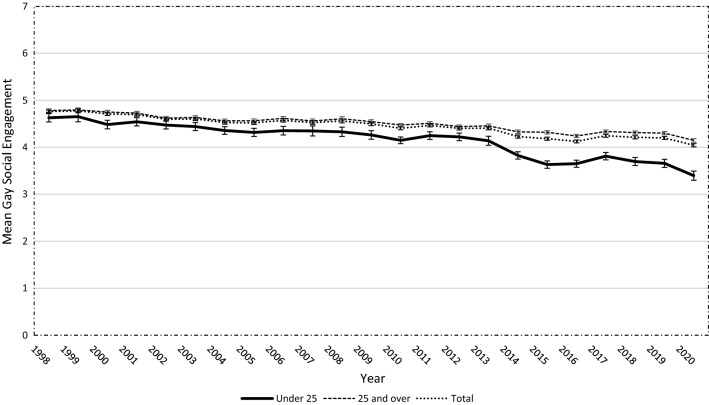
Mean Gay Social Engagement score by year and age group (*N* = 161,117)

Between 1998 and 2020, 166,936 survey responses were collected. Responses with missing data for the GSE measures were excluded (*N* = 5,819), leaving 161,117 survey responses in this analysis. Of the 5,819 excluded responses, 5242 (90.1%) were due to the GSE questions not being included in the survey conducted in one state (Queensland) during 2006–2009, with the remaining 577 (9.9%) being spread throughout the 20-year period with no more than 23 missing responses in a single round. The GSE data were negatively skewed with kurtosis of 2.61 and a skewness of -0.56. However, the sample was sufficiently large to use linear regression even if not normally distributed (Schmidt & Finan, 2018). Mean GSE in the sample declined from 1998 (*M* = 4.76, SD = 1.34) to 2020 (*M* = 4.04, SD = 1.63; *β* = −0.03, *p* < 0.001; Fig. 1). There was a steeper decline in GSE among younger participants (under 25 years) from 1998 (*M* = 4.63, SD = 1.39) to 2020 (*M* = 3.40, SD = 1.70; *β* = −0.05, *p* < 0.001). However, GSE also fell among older participants (aged 25 years and over) from 1998 (*M* = 4.78, SD = 1.33) to 2020 (*M* = 4.14, SD = 1.60; *β* = −0.03, *p* < 0.001).

### Cross-Sectional Analyses

In 1999/2000, 6,727 surveys were collected with 27 (0.4%) excluded due to missing data on GSE. Among the remaining 6,700, 1,237 (18.5%) were categorized as having low GSE and 5,463 (81.5%) categorized as having high GSE (*M* = 4.69, SD = 1.36). In 2009/2010, 9,081 surveys were collected with 33 (0.4%) excluded due to missing data on GSE. Among the remaining 9,048, 2,232 (24.7%) had low GSE and 6,816 (75.3%) had high GSE (*M* = 4.40, SD = 1.45). In the most recent rounds (2019/2020), 9,785 surveys were collected with 25 (0.3%) excluded due to missing data on GSE. Among the remaining 9,760, 3,439 (35.3%) had low GSE and 6,321 (64.8%) had high GSE (*M* = 4.04, SD = 1.62).

The results of the cross-sectional comparisons between GBM with low and high GSE in 1999/2000, 2009/2010 and 2019/2020 are shown in Table 1. At all timepoints, high GSE was associated at a bivariate level with older age, full time employment, testing for HIV in the previous twelve months and having 11 or more recent male sexual partners. In all timepoints, those who identified as bisexual or another sexual identity were significantly less likely to have high GSE compared to those who identified as gay. GSE and education were unrelated in 1999/2000 but university education was associated with higher GSE in the second and latest timepoints. In 1999/2000, low GSE was associated with CLAIC but the direction of this association reversed in the second timepoint (2009/2010), with high GSE associated with CLAIC. This was maintained in 2019/2020.

**Table 1:** Frequencies and unadjusted odds ratios in bivariate models predicting high gay social engagement at three timepoints between 1999 and 2020 (*N* = 25,508)

	1999/2000 (*N* = 6,700)			2009/2010 (*N* = 9,048)			2019/2020 (*N* = 9,760)		
	Low GSE *N* (%)	High GSE *N* (%)	OR (95% CI)	Low GSE *N* (%)	High GSE *N* (%)	OR (95% CI)	Low GSE *N* (%)	High GSE *N* (%)	OR (95% CI)
Age (M/SD)	34.1 (11.0)	35.3 (10.1)	**1.01 (1.01–1.02)**	33.1 (12.0)	36.2 (12.0)	**1.02 (1.02–1.03)**	36.9 (14.6)	38.2 (13.1)	**1.01 (1.00–1.01)**
University educated	369 (40.5)	1765 (44.0)	1.15 (1.00–1.33)	936 (43.7)	3217 (49.2)	**1.25 (1.13–1.38)**	1673 (48.8)	3873 (61.5)	**1.68 (1.55–1.83)**
Full time employment	758 (63.7)	3699 (69.2)	**1.28 (1.12–1.46)**	1342 (60.3)	4595 (67.9)	**1.39 (1.26–1.53)**	1962 (57.1)	4242 (67.2)	**1.54 (1.41–1.68)**
*Sexual identity*									
Gay	889 (72.9)	5053 (93.1)	**REF**	1609 (72.9)	6265 (92.4)	**REF**	2533 (74.2)	5674 (90.1)	**REF**
Bisexual	239 (19.4)	240 (4.4)	**0.18 (0.15–0.21)**	323 (14.6)	310 (4.6)	**0.25 (0.21–0.29)**	595 (17.4)	398 (6.3)	**0.30 (0.26–0.34)**
Other sexual identity	94 (7.7)	132 (2.4)	**0.25 (0.19–0.32)**	275 (12.5)	209 (3.1)	**0.20 (0.16–0.24)**	287 (8.4)	226 (3.6)	**0.35 (0.29–0.42)**
Received an HIV test^1^	597 (48.3)	3199 (58.6)	**1.51 (1.34–1.71)**	1062 (47.6)	4218 (61.9)	**1.79 (1.62–1.97)**	1825 (53.1)	4425 (70.0)	**2.06 (1.89–2.25)**
11 or more male sex partners^2^	252 (20.4)	1538 (28.2)	**1.53 (1.32–1.78)**	340 (15.2)	1615 (23.7)	**1.73 (1.52–1.96)**	415 (12.1)	1464 (23.2)	**2.20 (1.95–2.47)**
Any condomless anal intercourse with casual partners^2^	174 (14.1)	625 (11.4)	**0.79 (0.66–0.95)**	479 (21.5)	2032 (29.8)	**1.55 (1.39–1.74)**	982 (28.6)	2593 (41.0)	**1.74 (1.59–1.90)**
Any condomless anal intercourse with regular partners^2^	276 (22.3)	1332 (24.4)	1.12 (0.97–1.30)	709 (31.8)	3167 (46.5)	**1.86 (1.69–2.06)**	1340 (39.0)	3373 (53.4)	**1.79 (1.65–1.95)**
**Total**	**1,237**	**5,463**	–	**2,232**	**6,816**	**–**	**3,439**	**6,321**	**–**

^1^Previous 12 months

^2^Previous 6 months

Bold indicates statistical significance *p* < 0.05

The analyses at the latest timepoint using a wider range of measures are reported in Table 2. From the 9,760 participants included in the bivariate analyses, 1,500 participants had missing data on at least one variable and were excluded from the multivariable analysis. Comparing participants who were included (*n* = 8,760) and excluded (*n* = 1,500) from the multivariable analysis, excluded participants were less likely to be socially engaged (61.7% vs 65.3%, *χ*^*2*^ = 7.12, *p* = 0.008), were slightly older (39.7 vs. 37.4, *t* = −5.91, *p* < 0.001), less likely to be born in Australia (64.3% vs. 70.6%, *χ*^*2*^ = 19.79, *p* < 0.001), less like to identify as Anglo-Australian (63.2% vs. 68.3%, *χ*^*2*^ = 15.3, *p* < 0.001), more likely to identify as Aboriginal or Torres Strait Islander (4.8% vs. 3.5%, *χ*^*2*^ = 5.82, *p* = 0.016), less likely to be university educated (50.1% vs 58.2%, *χ*^*2*^ = 5.82), or in full time employment (58.9% vs. 64.5%, *χ*^*2*^ = 16.82, *p* < 0.001). Excluded participants were also more likely to identify as bisexual (14.3% vs 9.5%) or another sexual identity (7.3% vs 4.9%, *χ*^*2*^ = 47.53, *p* < 0.001), and more likely to have no recent male sex partners (25.4% vs. 16.5%, *χ*^*2*^ = 63.32, *p* < 0.001). No variables had a VIF of over 10 in the multivariable model.

**Table 2:** Frequencies and adjusted odds ratios in a multivariable regression model predicting high gay social engagement among GBM on data from 2019 to 2020 (*n* = 8,260)

	Not socially engaged *N* (%)	Socially engaged *N* (%)	OR (95% CI)	*p*	aOR (95% CI)	*p*
Age (Mean, SD)	37 (14.6)	38 (13.1)	1.01 (1.00–1.01)	** < 0.001**	1.01 (1.00–1.01)	** < 0.001**
*Country of birth*						
Overseas	911 (27.4)	1956 (31.7)	**REF**	**REF**	**REF**	**REF**
Australia	2412 (72.6)	4206 (68.3)	0.81 (0.74–0.89)	** < 0.001**	0.96 (0.85–1.08)	0.504
*Ethnicity*						
Anglo-Australian	2336 (67.9)	4257 (67.4)	**REF**	**REF**	**REF**	**REF**
Other	1103 (32.1)	2064 (32.7)	1.03 (0.94–1.12)	0.559	0.99 (0.88–1.08)	0.897
*Aboriginal or Torres Strait Islander*						
No	3301 (96.1)	6072 (96.4)	**REF**	**REF**	**REF**	**REF**
Yes	135 (3.93)	227 (3.60)	0.91 (0.74–1.14)	0.418	0.97 (0.74–1.27)	0.831
*Sexual identity*						
Gay	2533 (74.2)	5674 (90.1)	**REF**	**REF**	**REF**	**REF**
Bisexual	595 (17.4)	398 (6.3)	0.30 (0.26–0.34)	** < 0.001**	0.44 (0.38–0.52)	** < 0.001**
Other	287 (8.4)	226 (3.6)	0.35 (0.29–0.42)	** < 0.001**	0.61 (0.49–0.77)	** < 0.001**
*Education*						
No university education	1759 (51.3)	2422 (38.5)	**REF**	**REF**	**REF**	**REF**
University educated	1673 (48.7)	3873 (61.5)	1.68 (1.55–1.83)	** < 0.001**	1.41 (1.27–1.56)	** < 0.001**
*Employment*						
Not full time	1475 (42.9)	2071 (32.8)	**REF**	**REF**	**REF**	**REF**
Full time	1962 (57.1)	4242 (67.2)	1.54 (1.41–1.68)	** < 0.001**	1.13 (1.02–1.26)	**0.020**
*Relationship status*						
No sex with men	841 (25.1)	696 (11.3)	**REF**	**REF**	**REF**	**REF**
Regular and casual partners	850 (25.4)	2487 (40.3)	4.18 (3.69–4.74)	** < 0.001**	2.25 (1.88–2.69)	** < 0.001**
Monogamous	676 (20.2)	1787 (29.0)	2.97 (2.62–3.37)	** < 0.001**	2.26 (1.91–2.68)	** < 0.001**
Casual partners only	984 (29.4)	1196 (19.4)	2.50 (2.18–2.86)	** < 0.001**	1.50 (1.26–1.80)	** < 0.001**
*HIV testing history*^*1*^						
Not tested	1614 (46.9)	1896 (30.0)	**REF**	**REF**	**REF**	**REF**
Tested	1825 (53.1)	4425 (70.0)	2.06 (1.89–2.25)	** < 0.001**	1.24 (1.09–1.40)	**0.001**
*HIV status*						
HIV-negative not on PrEP	2004 (58.3)	3371 (53.3)	**REF**	**REF**	**REF**	**REF**
HIV-negative on PrEP	622 (18.1)	2075 (32.8)	1.98 (1.80–2.20)	** < 0.001**	1.35 (1.16–1.55)	** < 0.001**
HIV-positive	177 (5.2)	509 (8.1)	1.71 (1.42–2.05)	** < 0.001**	1.50 (1.21–1.87)	** < 0.001**
Unknown/untested	636 (18.5)	366 (5.8)	0.34 (0.30–0.39)	** < 0.001**	0.63 (0.53–0.76)	** < 0.001**
*Number of male sexual partners*^*2*^						
10 or fewer	3024 (87.9)	4857 (76.8)	**REF**	**REF**	**REF**	**REF**
11 or more	415 (12.1)	1454 (23.2)	2.20 (1.95–2.47)	** < 0.001**	1.13 (0.96–1.33)	0.154
*Condomless anal intercourse with casual partners (CLAIC)*^*2*^						
No CLAIC	2457 (71.5)	3728 (59.0)	**REF**	**REF**	**REF**	**REF**
Any CLAIC	982 (28.6)	2593 (41.0)	1.74 (1.59–1.90)	** < 0.001**	0.89 (0.77–1.03)	0.116
*Condomless anal intercourse with regular partners (CLAIR)*^*2*^						
No CLAIR	2099 (61.0)	2948 (46.6)	**REF**	**REF**	**REF**	**REF**
Any CLAIR	1340 (39.0)	3373 (53.4)	1.79 (1.65–1.95)	** < 0.001**	1.00 (0.88–1.13)	0.979
*Group sex*^*2*^						
No group sex	2592 (77.1)	3789 (61.0)	**REF**	**REF**	**REF**	**REF**
Some group sex	771 (22.9)	2419 (39.0)	2.14 (1.95–2.36)	** < 0.001**	1.20 (1.05–1.38)	**0.010**
*Recent injecting drug use*^*2*^						
No	3120 (97.4)	5789 (96.1)	**REF**	**REF**	**REF**	**REF**
Yes	84 (2.6)	236 (3.9)	1.52 (1.18–1.96)	**0.001**	0.94 (0.67–1.31)	0.696
*Used party drugs for sex*^*2*^						
No	3089 (89.8)	5043 (79.8)	**REF**	**REF**	**REF**	**REF**
Yes	350 (10.2)	1278 (20.2)	2.24 (1.97–2.53)	** < 0.001**	1.34 (1.31–1.79)	** < 0.001**
*Method of meeting partners*^*2*^						
Gay bar	370 (12.1)	1704 (31.1)	3.06 (2.71–3.46)	** < 0.001**	2.27 (1.96–2.63)	**0.001**
Mobile apps	1469 (46.2)	3387 (58.0)	1.54 (1.42–1.68)	** < 0.001**	0.79 (0.69–0.90)	**0.001**
Internet	811 (26.3)	1894 (34.4)	1.39 (1.26–1.52)	** < 0.001**	1.08 (0.95–1.22)	0.265
*Received STI diagnosis*^*1*^						
No	2580 (82.8)	4166 (71.5)	**REF**	**REF**	**REF**	**REF**
Yes	538 (17.3)	1660 (28.5)	1.91 (1.71–2.13)	** < 0.001**	1.10 (0.96–1.27)	0.163
**Total**	**3,439**	**6,321**				

^1^Previous 12 months

^2^Previous 6 months

Bold indicates statistical significance *p* < 0.05

At the multivariable level, high GSE was independently associated with older age (*p* < 0.001), being university educated (*p* < 0.001), in full time employment (*p* = 0.026), having either regular (*p* < 0.001) or casual (*p* < 0.001) male sex partners or both (*p* < 0.001), having received an HIV test in the previous 12 months (*p* < 0.001), engaging in group sex in the last 6 months (*p* = 0.007), using drugs for sex in the last 6 months (*p* < 0.001), meeting sex partners at gay bars (*p* = 0.001), or not meeting sex partners using mobile apps (*p* = 0.002). Compared to those who identified as gay, those who identified as bisexual or another sexual identity were significantly less likely to have high GSE (both *p* < 0.001). Compared to HIV-negative participants not on PrEP, those with an unknown or untested HIV status had lower GSE (*p* < 0.001), while HIV-negative participants on PrEP and HIV-positive participants had higher GSE (both *p* < 0.001). GSE was not independently associated with country of birth (*p* = 0.302), identifying as Anglo-Australian (*p* = 0.693), identifying as Aboriginal or Torres Strait Islander (*p* = 0.822)*,* number of male partners (*p* = 0.148), meeting partners via the internet (*p* = 0.224), recent CLAIC (*p* = 0.068), recent CLAIR (*p* = 0.876), injecting drug use (*p* = 0.468) or STI diagnoses (*p* = 0.251).

## Discussion

Three main findings emerged from this study. First, GSE declined gradually over the 20-year period with a steeper decline among young GBM. Second, GSE was found be associated with various demographic and behavioral outcomes relevant to HIV prevention throughout the 20-year period. This included condom use, HIV testing, and sexual behavior. Finally, GSE appears to remain relevant today as it has been consistently associated with testing and sexual behavior, and was associated with PrEP uptake in the latest timepoint. This has several implications for policy and health promotion for GBM.

Our finding that high GSE was associated with beneficial or protective factors such as HIV testing and PrEP uptake is consistent with previous research (Keen et al., 2020; Kuhns et al., 2017; Lauby et al., 2012; Scott et al., 2014; Zablotska et al., 2012). Those who have more gay friends and spend more time with them appear to access HIV-related health care more often than other GBM. There could be several potential mechanisms at work. There is evidence that awareness and willingness to take PrEP are associated with belonging to an LGBTQ social group (Zarwell et al., 2019) and knowing other PrEP users has been associated with PrEP uptake (Holt et al., 2019). Those who participate in GBM social networks may have increased exposure to people who are experienced in navigating HIV testing services or PrEP. This could facilitate the transfer of information and knowledge through direct communication or observation and modeling. In other populations, HIV testing has been found to be associated with an individual’s perception of HIV stigma and HIV testing norms within their social network (Johnson et al., 2021; Pullen et al., 2022). Theoretical frameworks to understand these associations have been developed (Johnson et al., 2010; Kaufman et al., 2014), but more work is needed to understand how factors within a social network, such as norms, stigma, and exposure to information, influence health behaviors and accessing health care.

At the earliest timepoint we considered (1999/2000), high GSE was associated with condom use and avoiding condomless sex. This is consistent with research early in the epidemic that suggested gay social networks drove the uptake of condoms for HIV prevention (Becker & Joseph, 1988; Hughes & Saxton, 2015; Kippax et al., 1992). Later in the epidemic (in the second and third timepoints we considered), participants with higher GSE were less likely to report consistent condom use with casual partners, reflected in other work conducted at the time (Fergus et al., 2009; Foster-Gimbel et al., 2020; Van De Ven et al., 2002). The middle timepoint of our analysis (2009/2010) reflects the period in which GBM increasingly utilized a range of partially effective risk reduction practices as alternatives to condoms, including serosorting, strategic positioning and withdrawal, practices that were first noted among small numbers of GBM in the late 1990s (Broady et al., 2020; Grov et al., 2015; Jin et al., 2007, 2009; Mao et al., 2006, 2011; Prestage et al., 2001; Suarez & Miller, 2001). There were significant arguments at the time about whether this shift away from consistent condom use represented complacency, optimism, or creativity in response to higher levels of HIV testing and treatment (Adam et al., 2005; Holt, 2014; Kippax & Race, 2003).

In the most recent timepoint (2019/2020), high GSE was associated with the highest level of CLAIC observed in our study period. This should be understood in the context of the introduction and adoption of PrEP, particularly through GBM’s inner city social networks (Holt et al., 2018, 2019; Zarwell et al., 2019) and increased viral suppression among HIV-positive GBM and knowledge of treatment as prevention (TasP) (Broady et al., 2020; De La Mata et al., 2015). Our analysis suggests that since the early 2000s men with high GSE have shifted their practices, first embracing condoms, then alternatives to condoms, and more recently PrEP. There continue to be increases in CLAIC among GBM with low GSE (from 14.1 in 1999/2000 to 28.6% in 2019/2020) but lower PrEP uptake (18.1% of participants with low GSE had taken PrEP compared to 32.8% of high GSE participants in 2019/2020). If norms concerning condom use have shifted due to a greater reliance on PrEP and TasP, it is a concern if there are groups, such as those with low GSE, who are more likely to engage in CLAIC but not as likely to use PrEP.

While higher GSE is associated with many protective factors, such as testing and PrEP use, those with high GSE scores were also more likely to report group sex and drug use for sex. These practices have been associated with increased HIV risk in high income countries like Australia (Bourne et al., 2015; Hammoud et al., 2017a, 2018; Mettey et al., 2003; Meunier & Siegel, 2018) and are also recognized features of metropolitan GBM’s social networks and cultures of drug use and sex partying (Bourne et al., 2015; Hurley & Prestage, 2009; Race et al., 2017). This demonstrates that GBM with high GSE can both engage in practices that may be considered “risky,” such as group sex or drug use for sex (sometimes referred to as “chemsex”), while also mitigating that risk through HIV testing or PrEP (Flores Anato et al., 2021; Hammoud et al., 2018; Hammoud, Vaccher, et al., 2018; Hibbert et al., 2019).

It is important to note that in the latest timepoint, a fifth of participants (22.9%) with low GSE engaged in group sex in the previous six months and a tenth (10.2%) used party drugs for sex, but also reported lower levels of PrEP use. Even if those with low GSE are comparatively less “risky” than high GSE participants, there are still some low GSE participants who engage in practices associated with HIV risk. Encouraging GBM with low GSE who engage in higher risk practices to incorporate strategies to mitigate their risk (e.g., taking PrEP, accessing harm reduction services) should be incorporated in HIV prevention interventions targeting GBM.

Interestingly, certain demographic characteristics have continued to be consistently associated with high GSE. Participants who were highly socially engaged with other gay men were typically older, well educated, and in full time employment. This suggests that there are intersections between socioeconomic status, geographic location and GSE. In Australia, many gay enclaves are in inner city areas that have a higher cost of living (Callander et al., 2020). GBM who lack the means to live in these areas may be less likely to be socially engaged as they are more distant from gay community spaces and events and therefore spend less time with gay men. Conversely, those who are motivated to be involved with gay community may make efforts to move to urban areas with greater access and exposure to community (Lewis, 2014). While it is unsurprising that identifying as gay is associated with high GSE, it is worth noting that there are still some gay men who are not highly engaged with other gay men, and conversely there are some bisexual men who are very engaged. Previous work has suggested that some bisexual men may feel superficially accepted in both gay and straight spaces, and may not necessarily feel like they belong in either setting, while other bisexual men feel accepted in all spaces (Dodge et al., 2012). However, this ambivalence to gay community is not unique to bisexual men as many gay men often do not feel connected to gay community (Holt, 2011; Winer, 2020). Our analysis showed that while identifying as gay is likely to be associated with high GSE, participants did not necessarily need to identify as gay to be highly engaged with other gay men, and those who were highly engaged with gay men were not necessarily gay-identified.

GSE has been gradually declining over the last 20 years and we have demonstrated that GSE is associated with both risk and protective factors against HIV. There are several potential reasons why GSE may be declining. As GBM have gained more legal and political rights, GBM have arguably become more able to express their sexual identities and live and socialize outside of gay enclaves and spaces (Holt, 2011; Zablotska et al., 2012). Additionally, the growth in online and mobile methods of meeting partners and social interaction has reduced GBM’s reliance on attending physical venues, which may have reduced time spent in person with other GBM (Card et al., 2018; Hull et al., 2016; Zablotska et al., 2012). This wider acceptance of sexual diversity means that GBM are more likely to have a variety of people in their friendship networks, including heterosexual people (Anderson & McCormack, 2016; Holt, 2011; Rawstorne et al., 2009; Rumens, 2011). Taken together, it is likely that GBM are now more likely to have friends other than gay men, and spend a smaller proportion of their time with gay men, leading to a decline in GSE. While the relative contribution of these factors to the decline in GSE has not been determined, the overall decline in GSE has implications for HIV prevention.

HIV diagnoses among GBM in Australia have been declining in recent years despite the decrease in GSE (Kirby Institute, 2020). It was possible that as GSE declined, HIV diagnoses would increase (due to less social support and pathways for HIV education and prevention) but this has not been the case. This may be because socially engaged GBM who were more likely to engage in CLAIC and be at risk of HIV infection were the group who benefitted most from increased HIV testing and PrEP in the most recent period, as well as effective HIV treatments promoting greater viral suppression among HIV-positive GBM. However, growing disparities between subpopulations of GBM have been identified as an ongoing challenge for HIV prevention and policy in Australia, such as GBM born in Asia becoming overrepresented in recent HIV infections (Gunaratnam et al., 2019). It is also unclear if the recent increases in HIV testing and PrEP that are concentrated in those with high GSE can be sustained if GSE continues to decline and GBM becomes less connected to networks of other GBM.

There are several limitations to this analysis. As the questionnaire is completed anonymously, we cannot identify and correct for participants who may have participated in more than one year. As there were differences between the demographic characteristics of excluded and included participants in the multivariate analysis, our results may be biased towards specific groups of GBM, such as younger men, those who identify as gay, and Anglo-Australian men who are university educated and in full-time employment. The two-item GSE measure used in this analysis is a simple measure that captures a limited view of the social lives of GBM. This is due to these questions being included in behavioral surveillance questionnaires that focus on HIV-related behaviors and do not ask in-depth questions about social networks. Despite being a simple and crude measure, GSE continues to remain associated with a variety of factors including HIV testing and sexual behavior, although the meaning of the measure may have changed over time. GSE does not capture social engagement with other LGBTQ+ people, particularly bisexual and pansexual men, who are likely to have their own sources of support that influence HIV and sexual health outcomes. Furthermore, the items in the GSE measure are relative measures, so participants with a large number of friends but a low proportion who are gay men would score lower on the GSE measure than someone with a small network completely comprised of gay men. If the absolute number of gay friends is more important in driving these associations than the relative proportion of gay friends, that would not be captured by the GSE measure. Combining a 0–3 item with a 0–4 item also overweights the 0–4 item in the GSE scale. Participants in this sample were recruited at gay venues and events so there is likely to be sampling bias towards GBM with higher GSE. However, there was diversity in GSE scores within this sample, and given we observed consistent, gradual changes over time, this suggests that GSE is lower and may have fallen even more among GBM less connected to gay social networks.

More work is needed to understand the mechanism by which GSE is associated with sexual behavior, HIV testing and PrEP uptake. Previous work concerning social networks and health behaviors outline potential mechanisms like direct communication through conversations (Kim et al., 2015; McDaid et al., 2021) or indirect influences such as conforming to perceived social norms, observation or role modeling (Kaufman et al., 2014; Mullen et al., 1987; Munro et al., 2007). However, we are unable to assess which of these mechanisms explains our findings with the measures we have available. Future work should consider the mechanism underlying the relationship between GSE and HIV-related behaviors, particularly if the mechanism is different in the contemporary period compared to when it was first investigated (Kaufman et al., 2014; Kippax et al., 1992), Our analysis showed that those with a university degree or full-time employment were more likely to have high GSE. This means some of the differences in HIV and sexual health related outcomes could be due to socioeconomic factors like education or income that may encourage health-seeking behaviors. More research is needed to understand the relationship between an individual’s social network, their sexual health literacy, and their health-seeking and HIV prevention-related behavior, particularly for GBM not well connected to other GBM. Beyond this, health promotion and interventions targeting sub-populations of GBM should incorporate understandings of how connection to other GBM can facilitate HIV testing, treatment, PrEP uptake and other relevant HIV prevention-related behaviors but also how these practices can be facilitated among those with little to no contact with other GBM.

### Conclusion

Social engagement with gay men has been and continues to be associated with sexual behavior and HIV testing among GBM, and more recently with PrEP use. However, declining levels of GSE pose challenges to health promotion targeting GBM. Further research is needed to understand how gay social networks impact on sexual health knowledge and behavior, and the implications of a shift away from predominantly gay social networks by GBM.

## Data Availability

Deidentified data used in this analysis will be shared to researchers after a request to the corresponding author.

## Code Availability

Code used in this analysis can be made available upon request to the corresponding author.
